# ﻿Morphological and phylogenetic analyses reveal two novel species of *Curvularia* (Pleosporales, Pleosporaceae) from southern China

**DOI:** 10.3897/mycokeys.120.156570

**Published:** 2025-07-29

**Authors:** Zhuan-Jun Guo, Mawuli Korsi Amenyogbe, Shi-Qiang Chen, Younes M. Rashad, Jian-Xin Deng, Huan Luo

**Affiliations:** 1 Department of Plant Protection, College of Agriculture, Yangtze University, Jingzhou 434025, China Yangze University Jingzhou China; 2 MARA Key Laboratory of Sustainable Crop Production in the Middle Reaches of the Yangtze River (Co–Construction by Ministry and Province), Yangtze University, Jingzhou 434025, China Arid Lands Cultivation Research Institute Alexandria Egypt; 3 Plant Protection and Biomolecular Diagnosis Department, Arid Lands Cultivation Research Institute (ALCRI), City of Scientific Research and Technological Applications (SRTA-City), New Borg El-Arab, Alexandria 21934, Egypt Yangtze University Jingzhou China; 4 College of Resources and Environment, Southwest University, Chongqing 400715, China Southwest University Chongqing China

**Keywords:** Dematiaceous hyphomycetes, morphology, multi-locus phylogeny, taxonomy, two new taxa

## Abstract

Members of *Curvularia* are of widespread clinical and agricultural importance, but species identification remains challenging due to morphological similarities and environmental variation. In this study, nine *Curvularia* strains were isolated from diseased leaves of *Nelumbonucifera* and *Nicotianatabacum* in Guangxi and Guizhou provinces, China. Morphological characteristics and phylogenetic analyses based on ITS, *GAPDH*, and *tef1-α* sequences revealed two novel species of *Curvularia* s. str., described here as *C.nuciferae***sp. nov.** and *C.tabaci***sp. nov.** This study enhances understanding of *Curvularia* taxonomy and expands the known species diversity.

## ﻿Introduction

The genus *Curvularia* encompasses species functioning as either pathogens or saprobes on various plant hosts, separated from the related genera and monographed by Manamgoda et al. (2015) and typified by *C.lunata*. Species of *Curvularia* predominantly target plants within the Poaceae family, posing significant threats to grasses and staple crops such as rice, maize, wheat, and sorghum (Pintos et al. 2019; van Vuuren et al. 2024; Adomako et al. 2025). However, they are not exclusively reported from Poaceae and also infect or are reported from a broad range of host families, including Amaryllidaceae, Cactaceae, Caricaceae, Convolvulaceae, Cyperaceae, Fabaceae, Moringaceae, Nyctaginaceae, and Portulacaceae (Marin-Felix et al. 2017a, 2020; Tan et al. 2018; Hernãndez-Restrepo et al. 2018; Cui et al. 2020; Crous et al. 2020; Safi et al. 2020). Beyond their agricultural importance, some *Curvularia* species cause opportunistic infections in humans. For instance, *C.chlamydospora* and *C.lunata* have been implicated in respiratory, skin, brain, and corneal infections, particularly in immunocompromised individuals (Carter and Boudreaux 2004; Madrid et al. 2014; Manamgoda et al. 2015; Kiss et al. 2020; Iturrieta-González et al. 2020). Some species have also been reported from aquatic habitats, including *C.lunata*, *C.pallescens*, *C.senegalensis*, and others (Pratibha et al. 2013).

Historically, *Bipolaris*, *Curvularia*, *Drechslera*, and *Exserohilum* were grouped under the genus *Helminthosporium* (Sivanesan 1987). Morphological identification within *Curvularia* and related genera has primarily relied on morphological characters (Manamgoda et al. 2011). However, distinguishing these genera can be challenging due to overlapping features influenced by environmental factors, host type, and growth medium (Tsuda et al. 1978; Dama Ram et al. 2024; Jayawardena et al. 2025). Key differences have been noted in their sexual and conidial traits. For example, the conidia are characterized by their distinctive curvature due to disproportionately larger intermediate cells and by a length usually less than 100 μm, a feature absent in *Bipolaris*, which exhibits evenly distributed curvature and longer conidia (Manamgoda et al. 2012; Marin-Felix et al. 2017a). Despite these morphological markers, identification based on morphology alone remains unreliable, as demonstrated by discrepancies in five species – *C.borreriae*, *C.catenulata*, *C.prasadii*, *C.trifolii*, and *C.tuberculata* – where molecular findings conflicted with traditional descriptions (Dama Ram et al. 2024).

The integration of molecular techniques, such as ITS, *GAPDH*, and *tef1-α* gene sequencing, has significantly advanced the classification of *Curvularia* species (Manamgoda et al. 2014; Tan et al. 2014; Marin-Felix et al. 2017b). These methods have provided a stable framework for resolving taxonomic complexities, revealing extensive genetic diversity within the genus (Madrid et al. 2014; Manamgoda et al. 2015). *GAPDH* has been suggested as a promising secondary barcode marker to distinguish *Curvularia* species (Ahmadpour et al. 2025). Through DNA sequence analyses, 29 species previously classified under *Bipolaris* have been reassigned to *Curvularia* s. str. (Manamgoda et al. 2012; Tan et al. 2014; Marin-Felix et al. 2020). The use of multigene phylogenetics has thus proven invaluable in differentiating species and validating classifications based on morphology. Recent research has broadened our knowledge of the diversity within the genus *Curvularia* (Jayawardena et al. 2025). Madrid et al. (2014) determined six major clades of *Curvularia* based on a combined sequence analysis of the ITS, LSU, *GAPDH*, and *tef1-α* gene regions. Kusai et al. (2015) revised Ellis’s (1971) taxonomic key, identifying four major species – *C.eragrostidis*, *C.geniculata*, *C.hawaiiensis*, and *C.lunata* – and incorporating three additional species: *C.australiensis*, *C.catenulata*, and *C.spicifera*. Ahmadpour et al. (2025) established a new distinct clade using phylogenetic analyses. Over the past six years, numerous new species have been identified worldwide (Hyde et al. 2017; Marin-Felix et al. 2017a, 2017b, 2020; Dehdari et al. 2018; Ferdinandez et al. 2021, 2023). The genus comprises 59 species from China listed in the USDA Fungal Databases (https://fungi.ars.usda.gov/; accessed 19 June 2025).

In this study, two novel *Curvularia* species, *C.nuciferae* and *C.tabaci*, were identified through a combination of morphological traits and multigene sequence analyses. This research adds to the expanding corpus of literature on *Curvularia* taxonomy and diversity.

## ﻿Materials and methods

### ﻿Isolation and morphological studies

*Curvularia* isolates were obtained from *Nelumbonucifera* leaves with brown spots collected from a pond in Liuzhou City (24°16′49″N, 109°21′85″E), Guangxi Province, in May 2023, and from *Nicotianatabacum* leaves with dry spots harvested from a plantation in Zunyi City (26°00′74″N, 106°44′36″E), Guizhou Province, in September 2022. The samples were placed in clean bags and transported to the laboratory for processing. Fungal isolation was conducted using two methods. In the first method, infected tissues were cut into small segments (2 × 2 mm, including both diseased and healthy portions) and placed on moist filter paper inside Petri dishes, which were incubated at 25 °C to induce sporulation (Zhao et al. 2023). Emerging fungal conidia were examined under a stereomicroscope, and a single conidium was isolated and transferred to fresh potato dextrose agar (PDA) plates (24 g/L Difco, 20 g/L agar) using a sterilized glass needle. For the second method, tissue segments were surface-sterilized by sequential immersion in 70% ethanol for 30 seconds, 2% sodium hypochlorite for 1 minute, and three washes in sterile water (Wei et al. 2022). These segments were then placed on PDA and incubated at 25 °C. Hyphal tips from the emerging fungal colonies were transferred to fresh PDA plates to obtain pure cultures. All purified cultures were stored as test-tube slants at 4 °C and in cryovials containing 20% glycerol at -80 °C. Specimens (holotypes) and their ex-types were deposited in the Fungi Herbarium of Yangtze University (YzU), Jingzhou, Hubei, China (434025). The newly introduced species were registered in MycoBank following the guidelines of Crous et al. (2004).

For cultural characterization, isolates were incubated on PDA and TWA (water agar supplemented with sterile wheat straws) at 25 °C for seven days in complete darkness. Colony size, texture, and color were documented, with color descriptions following Rayner’s (1970) color charts. To evaluate morphological features such as conidial size, shape, color, and sporulation patterns, fresh mycelia were transferred onto PCA (potato carrot agar) and TWA plates and incubated under the same conditions (Madrid et al. 2014; Guo 2016). After seven days, 80 randomly selected conidia were mounted in water, and digital images were captured using a Nikon ECLIPSE Ni–U microscope system (Nikon, Japan). Sporulation patterns were also documented at the same time.

### ﻿DNA extraction, PCR amplification, and sequencing

Genomic DNA was extracted from fresh mycelia cultured on PDA for 3–5 days using the CTAB method described by Watanabe et al. (2010). The ITS, *GAPDH*, and *tef1-α* gene regions were amplified using the primer pairs ITS5/ITS4 (White et al. 1990), gpd1/gpd2 (Berbee et al. 1999), and EF983/2218R (Schoch et al. 2009), respectively. Each PCR reaction consisted of a 25 μL mixture containing 21 μL of 1.1× Taq PCR Star Mix (TSINGKE), 2 μL of template DNA, and 1 μL of each primer. Amplification was performed using a BIORAD T100 thermocycler with the following conditions: an initial denaturation at 94 °C for 5 minutes, followed by 30 cycles of denaturation at 94 °C for 30 seconds, annealing at 55 °C (for ITS and *GAPDH*) or 53 °C (for *tef1-α*) for 30 seconds, extension at 72 °C for 1 minute, and a final extension step at 72 °C for 10 minutes. PCR products were verified by electrophoresis on 1% agarose gels. Successfully amplified products were purified and sequenced by TSINGKE Biotechnology (Beijing, China).

### ﻿Phylogenetic analyses

Preliminary sequence verification was conducted using BioEdit v.7.2.3 (Hall 1999), and sequences were assembled with manual adjustments using PHYDIT v.3.2 (Chun 1995). Relevant ITS, *GAPDH*, and *tef1-α* gene sequences of *Curvularia* strains were downloaded from the NCBI database (https://www.ncbi.nlm.nih.gov/genbank/). Sequence alignment and manual editing were performed using MEGA 11 (Tamura et al. 2021). Phylogenetic analyses were carried out on a combined dataset of ITS, *GAPDH*, and *tef1-α* gene regions using maximum likelihood (ML) and Bayesian inference (BI) methods. ML analysis was executed with RAxML v.7.2.8 (Stamatakis 2006), while the best-fit evolutionary model (GTR+I+G) for each partition was determined using MrModeltest v.2.3 (Posada and Crandall 1998) based on the Akaike Information Criterion (AIC). For BI analysis, two Markov Chain Monte Carlo (MCMC) chains were run for 1,000,000 generations, sampling every 100 generations. The first 25% of samples were discarded as burn-in, and a 50% majority-rule consensus tree was constructed to calculate posterior probability values. The resulting phylogenetic trees were visualized and edited in FigTree v.1.3.1 (Rambaut and Drummond 2010). Branch support values greater than 60% for ML bootstrap and 0.6 for posterior probability (PP) were indicated on the resulting phylogram.

## ﻿Results

### ﻿Phylogenetic analyses

**Figure 1.**
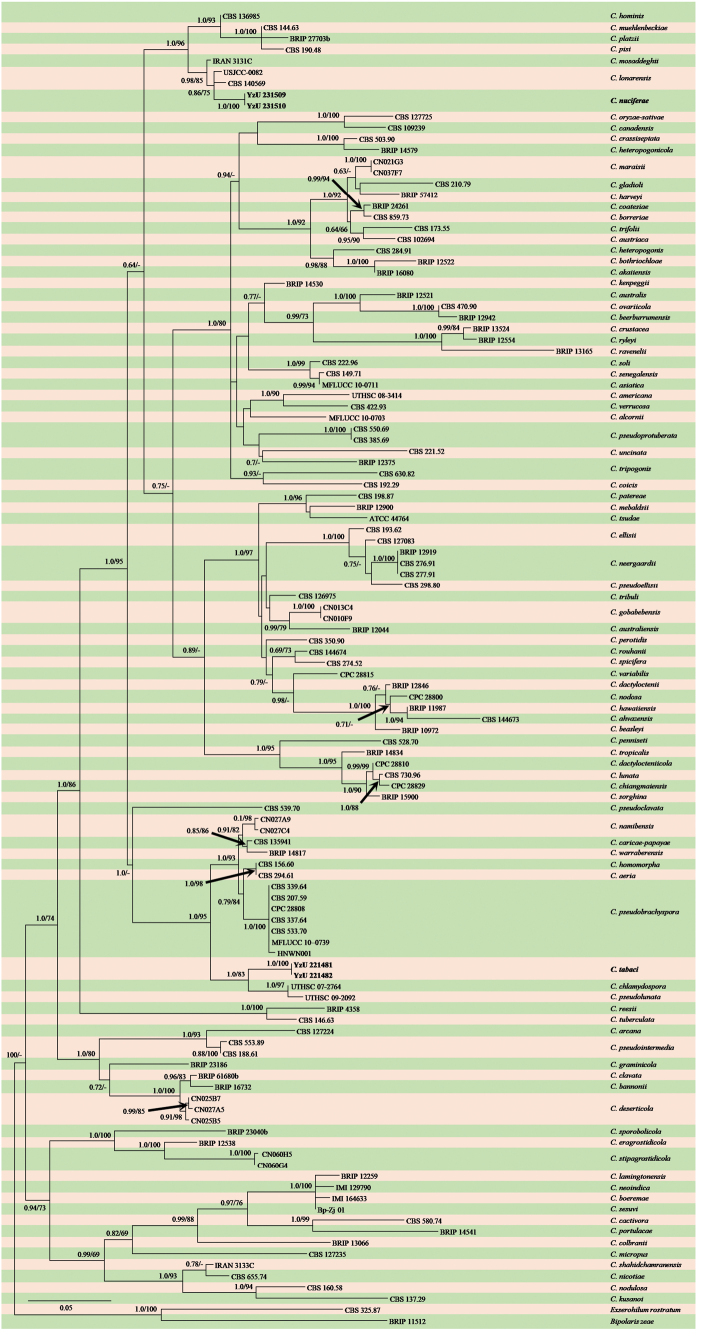
Phylogenetic tree of *Curvularia* based on the combined ITS, *GAPDH*, and *tef1-α* sequences. Bayesian posterior probabilities > 0.60 (PP) and maximum likelihood bootstrap support values > 60 (BS) are indicated at the nodes (PP/BS). The novel species are highlighted in bold.

Four representative strains, out of nine isolated *Curvularia* strains, were preliminarily identified based on morphological characterization. Following BLAST searches in GenBank, 114 relevant strains more closely related to the unidentified isolates were retrieved (Table 1). *Bipolariszeae* (BRIP 11512) and *Exserohilumrostratum* (CBS 325.87) were selected as the out-group. The combined dataset of three gene sequences (ITS, *GAPDH*, and *tef1-α*) comprised 1,532 bp (375 bp from ITS, 420 bp from *GAPDH*, and 737 bp from *tef1-α*), including alignment gaps. The phylogenetic analyses using maximum likelihood (ML) and Bayesian inference (BI) produced congruent tree topologies. The final consensus tree (Fig. 1) included RAxML bootstrap support (BS) and Bayesian posterior probability (PP) at the nodes. The clade containing YzU 231509 and YzU 231510 was phylogenetically close to *C.lonarensis* and *C.mosaddeghii* and was found to be independent with PP and BS values of 1.0/100%. Another clade, YzU 221481 and YzU 221482, was closely related to *C.chlamydospora* and *C.pseudolunata*, forming a distinct branch (1.0/100%). These results suggest that the present strains represent two new taxa.

**Table 1.** *Curvularia* strains and their GenBank accession numbers used in the phylogenetic analysis.

Species	Strains	Country	Host/Substrate	ITS	*GAPDH*	*tef1-α*
*Bipolariszeae*	BRIP 11512	Australia	*Zeamays*	KJ415538	KJ415408	KJ415454
*Curvulariaaeria*	CBS 294.61	Brazil	Air	HE861850	HF565450	–
*C.ahvazensis*	CBS 144673	Iran	*Zinniaelegans* rotten roots	KX139029	MG428693	MG428686
*C.akaiiensis*	BRIP 16080	India	Unknown	KJ415539	KJ415407	KJ415453
*C.alcornii*	MFLUCC 10-0703	Thailand	*Zea* sp.	JX256420	JX276433	JX266589
*C.arcana*	CBS 127224	Unknown	Unknown	MN688801	MN688828	MN688855
*C.asiatica*	MFLUCC 10-0711	Thailand	*Panicum* sp.	JX256424	JX276436	JX266593
*C.australiensis*	BRIP 12044	Australia	*Oryzasativa*	KJ415540	KJ415406	KJ415452
*C.australis*	BRIP 12521	Australia	*Sporobolus* sp.	KJ415541	KJ415405	KJ415451
*C.austriaca*	CBS 102694	Austria	Nasal cavity of patient with sinusitis	MN688802	MN688829	MN688856
*C.bannonii*	BRIP 16732	USA	*Jacquemontiatamnifolia*	KJ415542	KJ415404	KJ415450
*C.beasleyi*	BRIP 10972	Australia	*Chlorisgayana*	MH414892	MH433638	MH433654
*C.beerburrumensis*	BRIP 12942	Australia	*Eragrostisbahiensis*	MH414894	MH433634	MH433657
*C.boeremae*	IMI 164633	India	*Portulacaoleracea*	MH414911	MH433641	–
*C.bothriochloae*	BRIP 12522	Australia	*Bothriochloabladhii*	KJ415543	KJ415403	KJ415449
*C.cactivora*	CBS 580.74	Republic of Suriname	Member of Cactaceae	MN688803	MN688830	MN688857
*C.canadensis*	CBS 109239	Canada	Overwintered grass	MN688804	MN688831	MN688858
*C.caricae*-*papayae*	CBS 135941	India	*Caricapapaya*	HG778984	HG779146	–
*C.chiangmaiensis*	CPC 28829	Thailand	*Zeamays*	MF490814	MF490836	MF490857
*C.chlamydospora*	UTHSC 07-2764	USA	Toe nail	HG779021	HG779151	–
*C.clavata*	BRIP 61680b	Australia	*Oryzarufipogon*	KU552205	KU552167	KU552159
*C.coatesiae*	BRIP 24261	Australia	*Litchichinensis*	MH414897	MH433636	MH433659
*C.coicis*	CBS 192.29	Japan	*Coixlacryma*	JN192373	JN600962	JN601006
*C.colbranii*	BRIP 13066	Australia	*Crinumzeylanicum*	MH414898	MH433642	MH433660
*C.crassiseptata*	CBS 503.90	Nigeria	Plant material	LT631310	LT715882	MN688859
*C.crustacea*	BRIP 13524	Indonesia	*Sporobolus* sp.	KJ415544	KJ415402	KJ415448
*C.dactylocteniicola*	CPC 28810	Thailand	*Dactylocteniumaegyptium*	MF490815	MF490837	MF490858
*C.dactyloctenii*	BRIP 12846	Australia	*Dactylocteniumradulans*	KJ415545	KJ415401	KJ415447
*C.deserticola*	CN025B5	Namibia: Far East	*Stipagrostisciliata*	ON074982	ON355397	ON355358
	CN025B7	Namibia: Far East	*Stipagrostisciliata*	ON074983	ON355398	ON355359
	CN027A5	Namibia: Far East	*Stipagrostisciliata*	ON075008	ON355400	ON355361
*C.ellisii*	CBS 193.62	Pakistan	Air	JN192375	JN600963	JN601007
	CBS 127083	Australia	*Dactylocteniumaegyptium*	MN688805	MN688832	MN688860
*C.eragrostidicola*	BRIP 12538	Australia	*Eragrostispilosa*	MH414899	MH433643	MH433661
*C.gladioli*	CBS 210.79	Romania	*Gladiolus* sp.	HG778987	HG779123	–
*C.gobabebensis*	CN013C4	Namibia: Mirabib	*Stipagrostisciliata*	ON074797	ON355381	ON355347
	CN010F9	Namibia: Mirabib	*Stipagrostisciliata*	ON332848	ON355373	ON355344
*C.graminicola*	BRIP 23 186	Australia	Unknown	JN192376	JN600964	JN601008
*C.harveyi*	BRIP 57412	Australia	*Triticumaestivum*	KJ415546	KJ415400	KJ415446
*C.hawaiiensis*	BRIP 11987	USA	*Oryzasativa*	KJ415547	KJ415399	KJ415445
*C.heteropogonicola*	BRIP 14579	India	*Heteropogoncontortus*	KJ415548	KJ415398	KJ415444
*C.heteropogonis*	CBS 284.91	Australia	*Heteropogoncontortus*	JN192379	JN600969	JN601013
*C.hominis*	CBS 136985	USA	*Homosapiens*	sapiens	HG779011	HG779106
*C.homomorpha*	CBS 156.60	USA	Air	JN192380	JN600970	JN601014
*C.ischaemi*	CBS 630.82	New Zealand	*Ischaemumindicum*	JX256428	JX276440	–
*C.kenpeggii*	BRIP 14530	Australia	*Triticumaestivum*	MH414900	MH433644	MH433662
*C.kusanoi*	CBS 137.29	Japan	*Eragrostismajor*	JN192381	–	JN601016
*C.lamingtonensis*	BRIP 12259	Australia	*Microlaenastipoides*	MH414901	MH433645	MH433663
*C.lonarensis*	CBS 140569	India	Lonar lake	KT315408	KY007019	–
	USJCC–0082	Sri Lanka	*Saccharumofficinarum*	OQ275224	OQ269635	OQ332411
*C.lunata*	CBS 730.96	USA	Lung biopsy	JX256429	JX276441	JX266596
*C.maraisii*	CN037F7	Namibia: Far East	Soil	OR471647	ON355439	OR486044
	CN021G3	Namibia: Far East	*Stipagrostisciliata*	ON074886	ON355385	ON355351
*C.mebaldsii*	BRIP 12900	Australia	*Cynodontransvaalensis*	MH414902	MH433647	MH433664
*C.micropus*	CBS 127235	USA	*Paspalumnotatum*	HE792934	LT715859	–
*C.mosaddeghii*	IRAN 3131C	Iran	*Syzygiumcumini* leaf spot	MG846737	MH392155	MH392152
*C.muehlenbeckiae*	CBS 144.63	India	*Muehlenbeckia* sp.	HG779002	HG779108	–
*C.namibensis*	CN027C4	Namibia: Far East	*Stipagrostisciliata*	ON075010	ON355402	ON355363
	CN027A9	Namibia: Far East	*Stipagrostisciliata*	ON075009	ON355401	ON355362
*C.neergaardii*	BRIP 12919	Ghana	*Oryzasativa*	KJ415550	KJ415397	KJ415443
	CBS 276.91	Australia	Unknown	MN688806	MN688833	MN688861
	CBS 277.91	Australia	Unknown	MN688807	MN688834	MN688862
*C.neoindica*	IMI 129790	India	*Brassicanigra*	NR_158450	MH433649	MH433667
*C.nicotiae*	CBS 655.74	Algeria	Desert soil	KJ415551	KJ415396	KJ415442
*C.nodosa*	CPC 28800	Thailand	*Digitariaciliaris*	MF490816	MF490838	MF490859
*C.nodulosa*	CBS 160.58	USA	*Eleusineindica*	JN601033	JN600975	JN601019
***C.nuciferae***	**YzU 231509**	**China**	***Nelumbonucifera***	**OR888819**	**PP066825**	**2827452**
	**YzU 231510**	**China**	***Nelumbonucifera***	**OR888818**	**PP066826**	**2827459**
*C.oryzae-sativae*	CBS 127725	Argentina	*Oryzasativa*	MN688808	MN688835	MN688863
*C.ovariicola*	CBS 470.90	Australia	*Eragrostisinterrupta*	MN688809	MN688836	–
*C.stipagrostidicola*	CN060H5	Namibia: Reverse	*Stipagrostisciliata*	ON332838	ON355415	ON355368
*C.stipagrostidicola*	CN060G4	Namibia: Reverse	*Stipagrostisciliata*	ON332837	ON355414	ON355367
*C.patereae*	CBS 198.87	Argentina	*Triticumdurum* seed	MN688810	MN688837	MN688864
*C.penniseti*	CBS 528.70	Unknown	*Pennisetum* sp. seed	MN688811	MN688838	–
*C.perotidis*	CBS 350.90	Australia	*Perotisrara*	JN192385	KJ415394	JN601021
*C.pisi*	CBS 190.48	Canada	*Pisumsativum*	KY905678	KY905690	KY905697
*C.platzii*	BRIP 27703b	Australia	*Cenchrusclandestinum*	MH414906	MH433651	MH433669
*C.portulacae*	BRIP 14541	USA	*Portulacaoleracea*	KJ415553	KJ415393	KJ415440
*C.pseudobrachyspora*	CPC 28808	Thailand	*Eleusineindica*	MF490819	MF490819	MF490819
	CBS 207.59	Unknown	Unknown	MN688812	MN688839	MN688865
	CBS 533.70	Denmark	*Pennisetum* sp. seed	MN688813	MN688840	MN688866
	HNWN001	China	*Arecacatechu* leaf	MH516132	MH516133	MH516134
	CBS 337.64	USA	*Agropyronrepens*	MN688815	MN688842	MN688867
	CBS 339.64	USA	*Pennisetumglaucum*	MN688816	MN688843	MN688868
	MFLUCC 10–0739	Thailand	*Oryzasativa*	JX256443	JX276454	JX266603
*C.pseudoclavata*	CBS 539.70	Denmark	*Oryzasativa* seed	MN688817	MN688844	MN688869
*C.pseudoellisii*	CBS 298.80	Sudan	*Sorghumbicolor* seed	MN688818	MN688845	MN688870
*C.pseudointermedia*	CBS 553.89	Brazil	Cultivated pasture soil	MN688819	MN688846	MN688871
	CBS 188.61	Guadeloupe	Decaying grass	MN688820	MN688847	MN688872
*C.pseudolunata*	UTHSC 09-2092	USA	Nasal sinus	HE861842	HF565459	–
*C.pseudoprotuberata*	CBS 385.69	Canada	Soil under *Thujaoccidentalis*	MN688821	MN688848	MN688873
	CBS 550.69	Canada	Soil under *Pinusstrobus*	MN688822	MN688849	MN688874
*C.pseudorobusta*	UTHSC 08-3458	USA	Nasal sinus	HE861838	HF565476	–
*C.ravenelii*	BRIP 13165	Australia	*Sporobolusfertilis*	JN192386	JN600978	JN601024
*C.reesii*	BRIP 4358	Australia	Air	MH414907	MH433637	MH433670
*C.richardiae*	BRIP 4371	Australia	*Richardiabrasiliensis*	KJ415555	KJ415391	KJ415438
*C.rouhanii*	CBS 144674	Iran	Blighted leave of *Syngoniumvellozianum*	KX139030	MG428694	MG428687
*C.ryleyi*	BRIP 12554	Australia	*Sporoboluscreber*	KJ415556	KJ415390	KJ415437
*C.senegalensis*	CBS 149.71	Nigeria	Unknown	HG779001	HG779128	–
*C.sesuvi*	Bp-Zj 01	Unknown	*Sesuvium* sp.	EF175940	–	–
*C.shahidchamranensis*	IRAN 3133C	Iran	Soil	MH550084	MH550083	–
*C.soli*	CBS 222.96	Papua New Guinea	Soil	KY905679	KY905691	KY905698
*C.sorghina*	BRIP 15900	Australia	*Sorghumbicolor*	KJ415558	KJ415388	KJ415435
*C.spicifera*	CBS 274.52	Spain	Soil	JN192387	JN600979	JN601023
*C.sporobolicola*	BRIP 23040b	Australia	*Sporobolusaustralasicus*	MH414908	MH433652	MH433671
***C.tabaci***	**YzU 221481**	**China**	***Nicotianatabacum***	**OR888817**	**PP066824**	**OR818398**
***C.tabaci***	**YzU 221482**	**China**	***Nicotianatabacum***	**PP601257**	**PP779503**	**PP779504**
*C.tribuli*	CBS 126975	South Africa	*Tribulusterrestris* leaf	MN688825	MN688852	MN688875
*C.trifolii*	CBS 173.55	USA	*Trifoliumrepens*	HG779023	HG779124	–
*C.tripogonis*	BRIP 12375	Australia	Unknown	JN192388	JN600980	JN601025
*C.tropicalis*	BRIP 14834	India	*Coffeaarabica*	KJ415559	KJ415387	KJ415434
*C.tsudae*	ATCC 44764	Japan	*Chlorisgayana*	KC424596	KC747745	KC503940
*C.tuberculata*	CBS 146.63	India	*Zeamays*	JX256433	JX276445	JX266599
*C.uncinata*	CBS 221.52	Vietnam	*Oryzasativa*	HG779024	HG779134	–
*C.variabilis*	CPC 28815	Thailand	*Chlorisbarbata*	MF490822	MF490844	MF490865
*C.verrucosa*	CBS 422.93	Cuba	Air	MN688826	MN688853	MN688876
*C.warraberensis*	BRIP 14817	Australia	*Dactylocteniumaegyptium*	MH414909	MH433653	MH433672
*Exserohilumrostratum*	CBS 325.87	USA	*Homosapiens*	HE664035	LT715898	HE664082

CBS: Westerdijk Fungal Biodiversity Institute, Utrecht, the Netherlands; CPC: Culture Collection of Pedro Crous, housed at the Westerdijk Fungal Biodiversity Institute; IMI: International Mycological Institute, CABI–Bioscience, Egham, UK; IRAN: Iranian Fungal Culture Collection, Iranian Research Institute of Plant Protection, Tehran, Iran; MFLUCC: Mae Fah Luang University Culture Collection, Chiang Rai, Thailand; UTHSC: Fungus Testing Laboratory, Department of Pathology, University of Texas Health Science Center, San Antonio, Texas, USA; GUCC: Culture Collection at the Department of Plant Pathology, College of Agriculture, Guizhou University, China; YzU: Yangtze University, China; ITS: internal transcribed spacer region; *GAPDH*: partial glyceraldehyde-3-phosphate dehydrogenase gene; *tef1-α*: partial translation elongation factor 1-alpha gene.

### ﻿Taxonomy

#### ﻿ Curvularia nuciferae

Taxon classificationFungiPleosporalesPleosporaceae

J.X Deng & Z.J Guo sp. nov.

CB71E48B-6561-5EB9-9AB9-AB895D3652CD

851620

Fig. 2

##### Holotype.

China • Guangxi Province, Liuzhou City, 24°16′49″N, 109°21′85″E, from leaf spot of *Nelumbonucifera*, 8 June 2023 (**holotype**: YzU-H-2023138, ex-type YzU 231510 was stored in cryovials containing 20% glycerol at -80 °C).

##### Etymology.

The term “nuciferae” refers to the host species on which it was collected, *Nelumbonucifera*.

##### Description.

**Figure 2.**
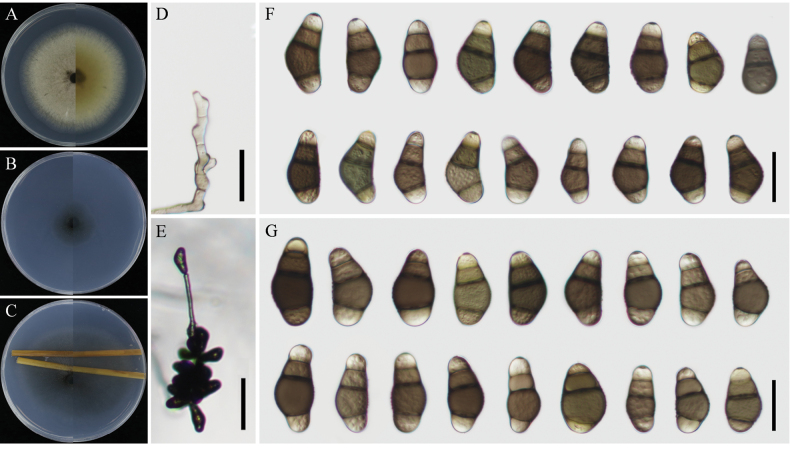
*Curvularianuciferae* sp. nov. (strain: YzU 231510). **A–C.** Colony phenotypes on PDA, WA, and TWA; **D.** Conidiophores; **E** Sporulation patterns; **F–G.** Conidia on TWA and PCA at 25 °C. Scale bars: 50 μm (**E**); 20 μm (**D, F, G**).

Colonies on PDA circular and flat shapes, vinaceous buff (no. 86) in the center with lighter margins on the plate. Reverse plates have olivaceous buff pigmentation with a diameter of 70–75 mm (Fig. 2). Colonies on TWA: Asexual morph—Hyphae 2.5–4 μm wide, subhyaline to brown, septate, branched. Conidiophores 105–328 × 4–6 μm (mean ± SD = 238.6 ± 68.2 × 5.1 ± 0.7 μm), solitary, flexuous, unbranched, septate, hyaline to brown, and lighter toward apex. Conidiogenous cells 9–15 × 4–7 μm (mean ± SD = 12.8 ± 2.1 × 5.2 ± 1 μm), terminal or intercalary, subcylindrical to slightly swollen, pale brown. Conidia 23–29(–32) × 12–16 μm (mean ± SD = 26.9 ± 2.6 × 13.6 ± 1.1 μm), smooth-walled, with the middle cells dark brown and the terminal ends paler, 2–3 septa. Additionally, the conidia were asymmetrically swollen and curved at the third cell from the base, with a protruding, darkened, and thickened hilum (2–3 μm). Sexual morph undetermined.

On PCA: Hyphae 2.5–4 μm wide, subhyaline to brown, septate, branched. Conidiophores 68–360 × 3–5 μm (mean ± SD = 222.3 ± 98.6 × 4.1 ± 0.9 μm), solitary, curved, ranging from nearly clear to dark brown, unbranched, erect, sometimes bent at the apex, thick-walled. Conidiogenous cells 9–16 × 4–6 μm (mean ± SD = 13 ± 2.5 × 5.2 ± 0.9 μm), terminal, proliferated sympodially, subcylindrical to irregular. Conidia 21–33(–40) × 10–18 μm (mean ± SD = 28.4 ± 4.6 × 14.1 ± 2.1 μm), slightly curved, smooth-walled, brown to dark brown, and swollen at the fourth cell from the base, 2–3 septate. No chlamydospores, microconidiation, or sexual morphs were observed.

##### Additional material studied.

China • Guangxi Province: Liuzhou City, on leaves of *Nelumbonucifera* (Nelumbonaceae, Proteales), 8 June 2023, collected by Z.J. Guo, S.Q. Chen & J.X. Deng (ex-type living culture: YzU 231510, ex-isotype: YzU 231509).

##### Notes.

*Curvularianuciferae* is phylogenetically close to *C.lonarensis* and *C.mosaddeghii* but can be distinguished from these species by its wider, lighter brown conidia, absence of bifurcate conidia, and larger conidia. Under the culturing conditions described by Sharma et al. (2016) (at 28 °C in darkness on PDA) and Ferdinandez et al. (2023) (at 25 °C with a 12 h/12 h photoperiod on PDA), conidia measuring 25–31(–35) × 12–18 µm and 22–30(–32) × 11–15 µm, respectively. *Curvularianuciferae* is similar to *C.lonarensis* but differs by having wider and lighter brown conidia and lacking the formation of bifurcate conidia (Ferdinandez et al. 2023). Additionally, it is easily distinguished from *C.mosaddeghii* by producing larger conidia (21–30(–34) × 9–13 µm) at 28 °C with a 12 h/12 h photoperiod (Heidari et al. 2018). After a nucleotide pairwise comparison, *C.nuciferae* can be readily differentiated from the other two related novel species based on ITS, *GAPDH*, and *tef1-α* gene regions, which have 0 bp and 3 bp differences in the ITS region, 8 bp and 8 bp in *GAPDH*, and 4 bp and 5 bp in *tef1-α* when compared with *C.lonarensis* and *C.mosaddeghii*, respectively. Strains YzU 231509 and YzU 231510 were placed in a single lineage within the same clade as *C.lonarensis* and *C.mosaddeghii*, supported by posterior probability and bootstrap values, but with sufficient genetic distance to be considered distinct species.

#### ﻿ Curvularia tabaci

Taxon classificationFungiPleosporalesPleosporaceae

J.X Deng & Z.J Guo sp. nov.

65135A3B-75DE-57E2-B29C-D603E6545896

851619

Fig. 3

##### Holotype.

China • Guizhou Province, Zunyi City, 26°00′74″N, 106°44′36″E, from leaf spot of *Nicotianatabacum*. 19 September 2022 (**holotype**: YzU-H-2022101, ex-type YzU 221481 was stored in cryovials containing 20% glycerol at -80 °C)

##### Etymology.

The epithet “tabaci” refers to the host species on which it was collected, *Nicotianatabacum*.

##### Description.

**Figure 3.**
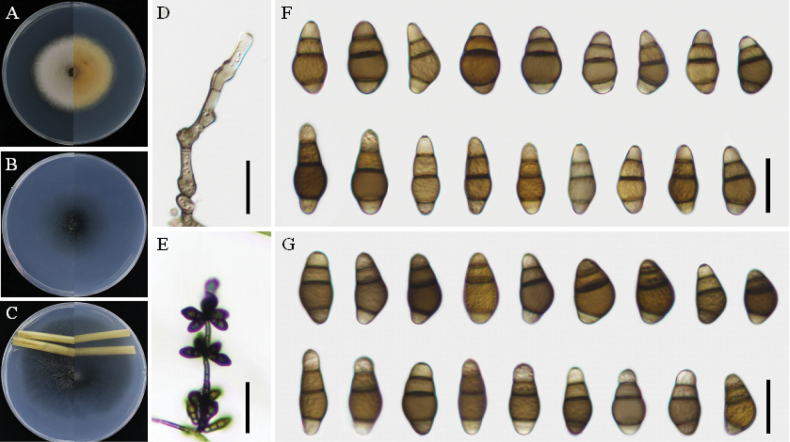
*Curvulariatabaci* sp. nov. (strain: YzU 221481). **A–C.** Colony phenotypes on PDA, WA, and TWA; **D.** Conidiophores; **E.** Sporulation patterns; **F–G.** Conidia on TWA and PCA at 25 °C. Scale bars: 50 μm (**E**); 20 μm (**D, F, G**).

Colonies on PDA circular, pale vinaceous grey (no. 115) structures with cottony mycelia in the center. The reverse side of the plate with ochreous pigmentation, with colonies measuring 51–56 mm in diameter (Fig. 3). Colonies on TWA: Asexual morph—Hyphae 2–4 μm wide, pale brown to brown, septate, branched. Conidiophores 52–247 × 4–6 μm (mean ± SD = 152.9 ± 69.6 × 5.2 ± 0.9 μm), straight to flexuous, septate, predominantly geniculate at the upper part, unbranched, pale brown to brown, paler toward apex. Conidiogenous cells 8–16 × 3–6 μm (mean ± SD = 10.8 ± 2.7 × 4.3 ± 1.7 μm), terminal or intercalary, proliferating sympodially, subcylindrical to slightly swollen. Conidia straight or slightly curved, 3-distoseptate, slightly constricted near the septa, brown to dark brown, apical and basal cells paler than the central cells. The basal third cell variably inflated, a punctate wall, measuring 23–29(–33) × 10–15 μm (mean ± SD = 26.2 ± 2.9 × 12.2 ± 1.7 μm). Hilum 2–3 μm, protruding, darker, and thickened. Sexual morph undetermined.

On PCA: Hyphae 2–4 μm wide, pale brown to brown, septate, branched. Conidiophores formed as solitary or flexuous, septate, pale brown to brown, becoming paler towards the apex, unbranched, 90–315 × 4–6 μm (mean ± SD = 222.2 ± 76.9 × 4.9 ± 0.7 μm). Conidiogenous cells terminal or intercalary, proliferating sympodially, and ranged from subcylindrical to irregular in shape, measuring 8–16 × 3–6 μm (mean ± SD = 12.8 ± 2.8 × 4.6 ± 1.2 μm). Conidia mostly curved, 2–4 celled, brown to dark brown, apical and basal cells paler than the central cells, and smooth-walled, measuring 20–26 × 10–13 μm (mean ± SD = 23.2 ± 2.2 × 11.8 ± 1.2 μm). Hilum 2–3 μm, protruding, darker, and thickened. No chlamydospores, microconidiation. Sexual morph undetermined.

##### Additional material studied.

China • Guizhou Province: Zunyi City, on leaves of *Nicotianatabacum* (Solanaceae, Lamiales), 19 September 2022, collected by Z.J. Guo, H. Luo & J.X. Deng (ex-type living culture: YzU 221481, ex-isotype: YzU 221482).

##### Notes.

Phylogenetic analysis revealed that *Curvulariatabaci* is closely related to *C.chlamydospora* and *C.pseudolunata* (Fig. 1). Pairwise DNA sequence comparison revealed that strains YzU 221481 and YzU 221482 exhibit identical sequences and are distinct from *C.chlamydospora* and *C.pseudolunata*. After a nucleotide pairwise comparison, *C.tabaci* differs from *C.chlamydospora* and *C.pseudolunata* by 5 and 4 bp in the ITS region and 13 and 9 bp in *GAPDH*, respectively. Furthermore, *C.chlamydospora* and *C.pseudolunata* have been reported as human pathogens in the USA (Madrid et al. 2014). Morphologically, *C.tabaci* (10–15 μm) differs from *C.chlamydospora* (7–12 μm) and *C.pseudolunata* (8–12 μm) by forming slightly wider, 2–4-celled conidia without observed chlamydospores or microconidiation. This branch represents a novel species more closely related to *C.chlamydospora* and *C.pseudolunata* (PP/BS = 1.00/83%) than to *C.aeria*, *C.caricae*-*papayae*, *C.homomorpha*, *C.namibensis*, *C.pseudobrachyspora*, and *C.warraberensis*.

## ﻿Discussion

In this work, nine fungal isolates with morphological characteristics similar to *Curvularia* were obtained from plant leaves belonging to the Nymphaeaceae and Solanaceae families. The identification of *Curvularia* species was frequently insufficient based on micromorphology, as many species have overlapping conidial dimensions (Manamgoda et al. 2015; Tan et al. 2018). In this study, it was found that standard conditions are quite important for *Curvularia* taxonomy based on morphological characteristics. Standardized morphological criteria should be adopted to minimize taxonomic inconsistencies arising from variations in temperature and substrates. To minimize potential misidentification of *Curvularia* species, we followed recommendations by Madrid et al. (2014) and Guo (2016), using PCA and TWA media for seven days at 25 °C. These media effectively promoted typical morphological characteristics. To enhance species identification within *Curvularia* and related genera, molecular techniques have become increasingly essential. According to Ram et al. (2024), approximately 25% of *Curvularia* species identified through morphological analysis are inconsistent with molecular findings – an issue that needs to be addressed through the exploration of more powerful gene fragments.

Previous studies have shown that the ITS region, while lacking clear discriminatory power, remains necessary for *Curvularia* identification (e.g., Manamgoda et al. 2014, 2015; van Vuuren et al. 2024). In the individual alignments of the three loci, the two new species – *Curvularianuciferae* and *C.tabaci* – were clearly distinguished from their related species using the *GAPDH* gene. In addition, the *tef1-α* region is recommended as a barcode for *Curvularia*, although sequence information for some species remains unavailable (Manamgoda et al. 2015). The *GAPDH* gene shows high resolution in distinguishing closely related species within *Curvularia*, which is consistent with previous research (Manamgoda et al. 2015; Ferdinandez et al. 2021; Ahmadpour et al. 2025). Currently, a polyphasic approach – integrating morphological characteristics with molecular phylogenetic data – is used to characterize and differentiate these taxa.

This study identified two new species, *Curvularianuciferae* and *C.tabaci*, using both morphological traits and multi-locus phylogenetic analyses of ITS, *GAPDH*, and *tef1-α* gene regions. The isolates were collected from *Nelumbonucifera* (lotus) and *Nicotianatabacum* (tobacco) in Guangxi and Guizhou provinces, respectively. This research contributes to the expanding diversity of *Curvularia* by describing two novel species. In studies on the diversity of endophytic fungi in lotus (*Nelumbonucifera*), Chen and Kirschner (2018) reported 33 genera, with 71% parasitizing leaves, but did not detect *Curvularia*. However, *C.lunata* has been found on lotus leaves in earlier studies (Cui and Sun 2012) and in Thailand (Techaoei et al. 2018). In tobacco production, *Curvularia*, along with *Alternaria*, *Aspergillus*, *Cladosporium*, and *Penicillium*, negatively affects both yield and quality (Chen et al. 2017, 2021). Additionally, notable species such as *C.australiensis*, *C.clavata*, and *C.trifolii* have been reported in tobacco (Chen et al. 2017; He et al. 2021; Motlagh et al. 2023). Hence, this research contributes to the expanding diversity of *Curvularia* by describing two novel species: *C.nuciferae* from *N.nucifera* and *C.tabaci* from *N.tabacum*.

## Supplementary Material

XML Treatment for Curvularia nuciferae

XML Treatment for Curvularia tabaci

## ﻿Additional information

### Use of AI

No use of AI was reported.

### Author ORCIDs

Zhuan-Jun Guo https://orcid.org/0009-0006-5760-4536

Mawuli Korsi Amenyogbe https://orcid.org/0000-0002-3180-7726

Shi-Qiang Chen https://orcid.org/0009-0003-7533-921X

Younes M. Rashad https://orcid.org/0000-0002-7702-8023

Jian-Xin Deng https://orcid.org/0000-0001-7304-5603

Huan Luo https://orcid.org/0000-0002-5503-2507

### Data availability

All of the data that support the findings of this study are available in the main text.
